# Exogenous lipoid pneumonia (ELP): when radiologist makes the difference

**Published:** 2016-05-16

**Authors:** G Rea, F Perna, G Calabrese, A Molino, T Valente, A Vatrella

**Affiliations:** 1. Department of Diagnostic Imaging, Monaldi Hospital, Naples, Italy; 2. Respiratory Medicine Division, Department of Clinical Medicine and Surgery, Federico II University, Naples, Italy; 3. Department of Medicine and Surgery, University of Salerno, Salerno, Italy

**Keywords:** Exogenous Lipoid pneumonia(ELP), High Resolution Computed tomography (HRCT), Bronchoalveolar lavage (BAL)

## Abstract

Lipoid pneumonia is an uncommon disorder characterized by accumulation of lipid components into the interstitial and alveolar compartment. The usual classification distinguishes endogenous and exogenous and acute or chronic forms, related to the type of fats, the amount of damage and the time of exposure. We describe a case of exogenous lipoid pneumonia by inhalation of vaseline used for cleaning of the tracheostoma in a 63-year-old female, presenting as cough, worsening dyspnea in few weeks. The diagnosis was finally established with a re-evaluation of BAL with specific staining for lipids, revealing the presence of foamy macrophages lipids rich, according to HRCT findings.

## INTRODUCTION

Lipoid pneumonia (LP) can be defined the result of pulmonary accumulation of lipid material within intra-alveolar macrophages of the lungs. The incidence is not clearly estimable for poor clinical symptoms and signs linked to inhalation and heterogeneous clinical, radiological and pathological features; furthermore it can be underdiagnosed or misdiagnosed with other alterations (bacterial pneumonia, lung tumor, tuberculosis, organizing pneumonia etc).

However some data from past autopsy series have established an incidence of about 1–2.5% [1].

The most common classification distinguishes endogenous and exogenous forms. It is also classified as either acute or chronic, depending on the amount of damage and the time of exposure [2]. We describe a case of exogenous lipoid pneumonia presenting as cough, dyspnea progressing over a few weeks.

## CASE REPORT

**Fig 1.**
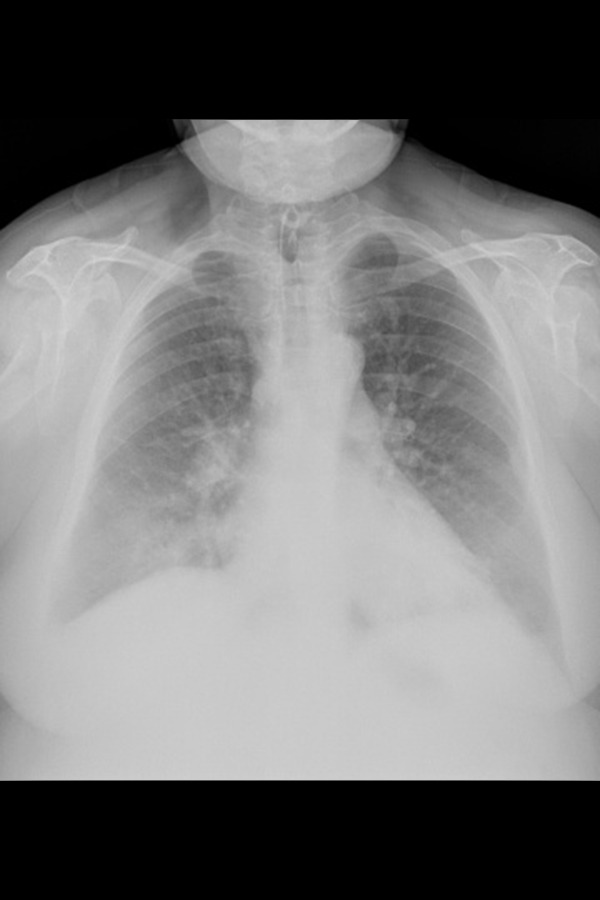
PA CXR : opacity poorly defined in lower right thoracic field near hemi-diaphragm.

**Fig 2 a–b):**
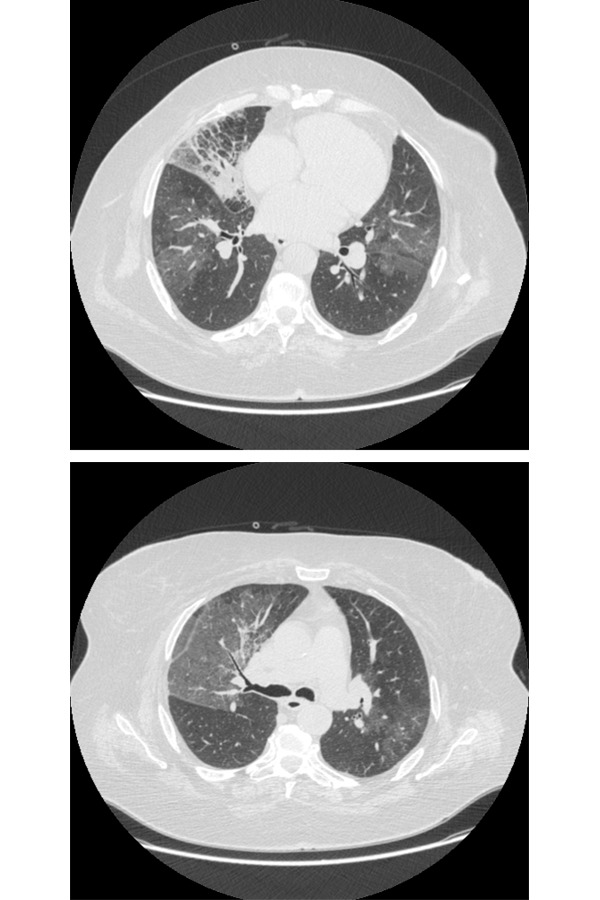
HRCT scan. Multiple extended GGO areas in both lung with middle lobe consolidation.

A 63-year-old female former smoker was referred to our Department with cough, low-grade fever (maximum temperature reached 37.5 ° C), worsening of dyspnea for several weeks. The patient stated that she had not been previously exposed to environmental allergens and she was being treated for systemic hypertension, diabetes mellitus second type, hypercholesterolemia. Clinical recently history showed a total laryngectomy for a cancer of the larynx, and stenosis and aortic regurgitation. On admission, the patient had cough with mucous-purulent sputum, dyspnea for mild efforts and showed signs of respiratory failure; in fact she presented arterial blood hypoxemia with normal levels of CO_2_ and severe desaturation to six minutes walking test (6MWT). The patient completed the test for the duration of six minutes, going 396 meters. However, the saturation down from an initial value of 90% to a value of 85% and for this reason, she had moreover mild cyanosis. Physical examination revealed hypomobility of ribcage, reduced breath sounds and crackles “velcro sounds” in lower lung fields. Blood tests showed leukocytosis (12.82×103/uL) with neutrophilia and elevated erythrocyte sedimentation rate (ESR 5 mm1 hour), while CRP was within the limits (0.5 mg/dl). Chest X-ray (CXR) showed increase of pulmonary hila, more evident to right with opacity poorly defined in cardiophrenic right angle. Also visible a slight increase right emidiaphragm (Fig 1). For the poorly specific signs of CXR an HRCT examination of the chest was made in external center. HRCT scans showed multiple extensive areas of ground glass opacities (GGO) in both lung with an irregular triangular consolidation with pneumonia like pattern in middle lobe. Also visible GGO near the consolidation (Fig 2a, b).

**Fig 3 a–b:**
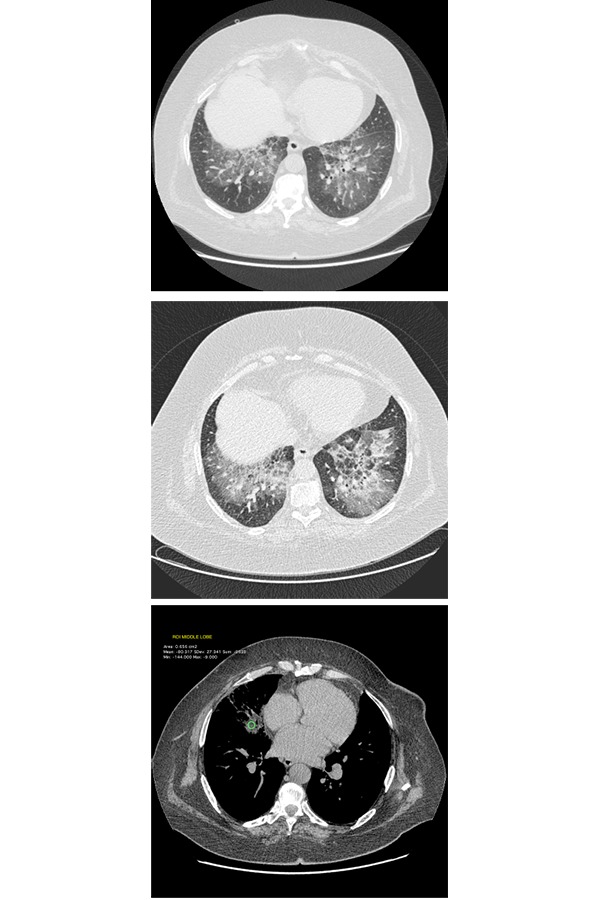
HRCT scan (lung windows) and c (mediastinal window). Geometric areas with extensive “patchy” appearance of GGO, like crazy paving with fine contextual reticulation with peribronchial and hilar prevailing distribution in the upper lobes, middle lobe, with great extension in both lower lobes. The results of measurements made inside middle lobe consolidation showed density values consistently negative (− 80 HU) expression of lipoid pneumonia (mediastinal window).

For this HRCT pattern a diagnosis of pneumonia was assumed. The patient begins treatment with a quinolone (levofloxacin, 500 gr/die), macrolide (clarithromycin 500 gr bid) and corticosteroid (betamethasone 1.5 mg/die) for ten days. During hospitalization, the patient underwent bronchoscopy, which revealed accumulation of mucous-purulent secretions, intense hyperemia and congestion of the tracheo-bronchial mucus membranes. Secretions were aspirated for cytological and microbiological study and broncho-alveolar lavage fluid was collected for immunological study. No microorganism was isolated by bacteriological examination and not malignant cells were found, but cytological analysis of broncho-alveolar lavage fluid showed neutrophils (75%) with increase of the CD4 / CD8. After clinical improvement and normalization of blood tests, the patient was discharged with indication practicing antibiotics (azithromycin, 500 mg once a day for 3 days a week, for 4 weeks), corticosteroids therapy (prednisone, 12.5 mg daily) and subsequent ambulatory monitoring. At the time of control, after three months, the patient reported persistent dyspnea. Another HRCT of the chest was performed to better highlight the previous GGO and consolidation. Unfortunately new exam showed no improvement of findings revealed on the previous examination, and also extensive areas with “patchy” GGO pattern with reticulation inside (crazy paving pattern), and the persistence of areas of consolidation with air bronchogram in middle lobe, and also new evidence of “crazy paving pattern” was visible in small areas of lingula and lower lobes. A ROI (region of interest) positioned within consolidations, showed negative density values (−45 HU/ −60 HU), highly suggestive of lipoid pneumonia (Fig 3a-b-c).

**Fig 4:**
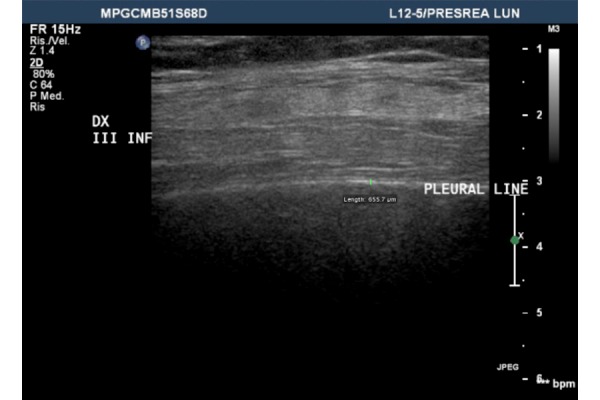
Lung Ultrasound (LUS): High frequency transducer (12 Mhz) linear probe positioned on anterior right axillary line (middle lobe). Normal hyperechoic pleural line and complete absence of horizontal reverberations (A-lines) and also absence of ring down artifacts (B-lines) and comet tails.

Lung ultrasound examination (Fig. 4) of the middle anterior region of right hemithorax (middle lobe), showed normal hyperechoic pleural line, but complete absence of horizontal reverberations (A-lines) and also absence of ring down (B-lines) and comet tail artifacts. These findings were not-specific, however they could be related to an alteration of the balance fluid/ gas in the interstitial –alveolar compartment [3].

**Figura 5):**
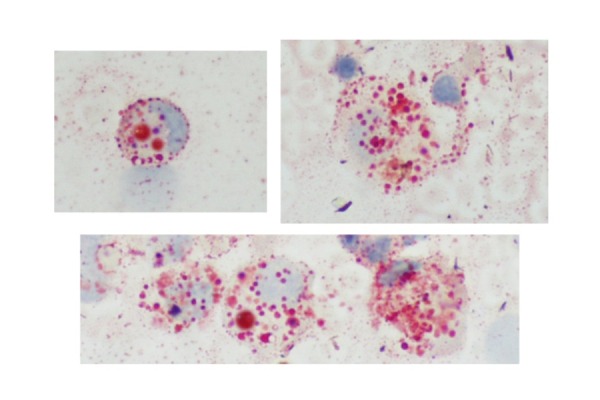
Alveolar macrophages recovered by bronchoalveolar lavage. The cytoplasm is full of red-staining cytoplasmic vacuoles filled with lipid that displace the nucleus to the periphery (oil red O stain, original magnification ×400).

For HRCT evidence, radiologist recommended a reevaluation of BAL with specific staining and coloration, which showed the presence of fat-laden macrophages that are found in lipoid pneumonias, therefore confirming the HRCT diagnosis (fig 5).

Investigating on the possible use of oily substances, patient reported the frequent use of vaseline oil to clean tracheal cannula after laryngectomy, moisturizing the area around the stoma and causing recurrent oil aspiration, whose behavior would explain exogenous lipoid pneumonia (ELP), while the immune-mediated response secondary to inflammatory phenomena repeated over time, is the basis of HRCT findings consistent with possible organizing pneumonia (OP). The patient was therefore treated with oxygen therapy, antibiotics (piperacillin / tazobactam, 2.25 g bid) and prednisone (double dose compared to the previous treatment: 25 mg daily for six months). Patient was instructed to immediately suspend the use of oily substances to lubricate the tracheal cannula. HRCT performed two months later, showed a persistence of the findings described above; in particular ELP located in the middle lobe, and OP-like alterations near fat density consolidations.

## DISCUSSION

Exogenous lipoid pneumonia (ELP) is an uncommon pulmonary disorder resulting from chronic aspiration or inhalation of mineral oil or a related material into the distal lung. It was described first time by Laughlen in 1925 and frequently observed between the 1940s and 1950s, for use of nasal medications containing petroleum (paraffin oil instillation) used for the treatment of tuberculosis [4, 5]. In the endogenous form, also called “cholesterol or golden pneumonia”, cholesterol and its esters derived from the destruction of alveolar cell wall and tissue repair during a suppurative process or post-obstructive bronchial condition, then producing lipoid pneumonias [6]. It may be associated with organizing pneumonia, malignancies, sarcoidosis, fat embolism, Gaucher and Niemann Pick disease [7]. The exogenous form originates from inhalation or exposure to oil (medications, laxatives, food, X-ray contrast media). Other less common causes are reported: egg yolk, use of petroleum jelly (Vaseline, Vicks TM) and lip balm [8]. Most common risk factors for LP are anatomical abnormalities of pharynx and esophagus, hiatal hernia, gastro-esophageal reflux, neuro-muscolar and psychiatric disorders [9]. Pathological findings shows lipoid pneumonia as a chronic foreign body reaction to fat, characterized by lipid-laden macrophages in lungs. The development of parenchymal abnormalities in lipoid pneumonia is dependent on the type, amount, frequency, and length of time of aspirated or inhaled oils or fats. Mineral oil (a mixture of inert, long-chain, saturated hydrocarbons obtained from petroleum) and vegetable-based oils tend to cause minimal to mild inflammatory reactions. The intra-alveolar oils can coalesce in the alveoli and become encapsulated by fibrous tissue, resulting in a nodule or mass. Conversely, animal fats are hydrolyzed by lung lipases into free fatty acids that trigger a severe inflammatory reaction that manifests as focal edema and intra-alveolar hemorrhage. Fatty acids either remain in the alveolar spaces or are phagocytized by macrophages that then migrate to the interlobular septa, as in our cases producing a severe crazy paving pattern, for the reactivity of the inflammatory lung cells to lipoids infiltration. Regardless of location, the inflammatory response can destroys the alveolar walls and the interstitium, and the resultant fibrosis can occasionally progress to end-stage lung disease (honeycombing pattern). This may explain the variability of the clinical manifestations and of the morphological patterns detected by HRCT scans. Clinical course of the disease appears variable from asymptomatic form to life-threatening disease. The most common presentation includes chronic cough, often productive and dyspnea. Other less frequent symptoms are chest pain, hemoptysis, weight loss, fever, extra-thoracic symptoms (abdominal pain, dysphagia, vomiting). These patients may present a restrictive, obstructive or mixed pattern on PFT [10]. Diagnosis of LP comes out from the integration of history, clinical examination, radiological and when possible by cytological findings. CXR has a low accuracy because LP may present with different nonspecific HRCT patterns (diffuse consolidations, reticular or nodular pattern, unilateral-bilateral nodular opacities) and distribution (lower and middle lobes are most commonly affected, although the location in the upper lobes was observed) [11]. Chest HRCT usually shows airspace consolidations, ground glass opacities, crazy paving pattern, interlobular septal thickening, mass-like lesions [12]. The septal thickening is caused by infiltration of lipid-laden macrophages and subtle interstitial inflammation. Multiple aspirations may result in GGO and crazy paving pattern and also may be associated to organizing pneumonia, a non-specific response to various forms of lung injury. The presence of consolidations within negative densities values (−30 / −150 HU) is highly suggestive of fat density. For a proper HRCT diagnosis, is correct to do a suitable sampling with a ROI (region of interest) positioned within consolidation, with maximum attention to avoid sampling liquid and or air [13]. CT-PET usually shows significant high SUV (standard uptake value) in these diseases, and for this reason it should be avoided as diagnostic investigation for the high value of sensitivity, but very low specificity, determining great clinical and diagnostic confusion with incorrect management of the patient [14]. The identification of lipid laden macrophages in broncho-alveolar lavage is a marker that confirms the diagnosis strongly suspected by chest HRCT. The disease is frequently associated to inflammatory cellular infiltration in acute /early stage of disease and organizing pneumonia and fibrosis in late stage. The presence of foamy macrophages with large cytoplasmic vacuoles is frequently seen in exogenous form [15]. In case of uncertain diagnosis, transbronchial biopsy (TBNA) or lung surgical biopsy by video-assisted thoracic surgery (VATS) may be indicated. In literature, to date, there are currently no defined data regarding the most appropriate therapy. Treatment is generally conservative. Key element is to identify and remove the cause of damage: oil agent. The use of corticosteroids remains a controversial practice that is generally performed in cases of severe lung injuries [16]. In our case, the elimination of the use of vaseline, oxygen therapy and corticosteroid treatment resulted in a significant improvement of reversible component of lipoid pneumonia, but not in full resolution because of evolution to crazy paving pattern “OP-like” [17]. In our opinion the failure to regression of the lesions can be attributed to GGO pattern characterized by fine fibrotic networks inside, with interlobular and intra-lobular thickening (crazy paving), expression of fine fibrosis rather than potentially reversible alveolitis, especially after suitable corticosteroid therapy. In conclusion, lipoid pneumonia is a rare, often underdiagnosed, disease due to the heterogeneity of clinical and radiological manifestations. The disease should be suspected on the basis of a careful history, thorough assessment of the clinical and radiological features, which can mimic many other diseases, and should be confirmed through the close collaboration between clinicians, radiologists and pathologists with a multi-disciplinary approach.
